# Melioidosis in South Asia: Scientific Advances, Regional Challenges and One Health Perspectives—An Integrated Research Synthesis from the 4th South Asian Melioidosis Congress

**DOI:** 10.12688/wellcomeopenres.27378.1

**Published:** 2026-08-21

**Authors:** Chiranjay Mukhopadhyay, Chimanjita Phukan, Saurav Jyoti Patgiri, Harpreet Kaur, Bart J Currie, David AB Dance

**Affiliations:** 1 Department of Microbiology, Kasturba Medical College Manipal, Manipal, Karnataka, 576104, India; 2 Department of Microbiology, Assam Medical College and Hospital, Dibrugarh, Assam, 786002, India; 3 ICMR - National Institute of Health Research, Dibrugarh, Assam, 786001, India; 4 Division of Communicable Diseases, Indian Council of Medical Research, New Delhi, Delhi, 110029, India; 5 Global and Tropical Health Division, Menzies School of Health Research and Charles Darwin University, Darwin, Northern Territory, 0810, Australia; 6 Division of Medicine, Royal Darwin Hospital Department of Infectious Diseases, Casuarina, Northern Territory, NT 0810, Australia; 7 London School of Hygiene and Tropical Medicine Faculty of Infectious and Tropical Diseases, London, England, WC1E 7HT, UK; 8 Lao-Oxford-Mahosot Hospital-Wellcome Trust Research Unit, Vientiane, 01000, Lao People's Democratic Republic

**Keywords:** Melioidosis, Burkholderia pseudomallei, South Asia, One Health, Diagnostics, Genomics, Tropical Diseases, Surveillance

## Abstract

**Background:**

Melioidosis, caused by *Burkholderia pseudomallei*, is a severe tropical infection with diverse clinical manifestations and high mortality if untreated. Although endemic in Southeast Asia and northern Australia, its burden is increasingly recognized in South Asia, where the disease remains underdiagnosed and is frequently misclassified as tuberculosis or other infections. This review synthesizes the contemporary scientific evidence and expert consensus emerging from the 4th South Asian Melioidosis Congress held in Guwahati, Assam, India, in November 2025.

**Methods:**

We conducted an integrated narrative synthesis of the scientific content presented at the Congress, including plenary lectures, oral and poster presentations, panel discussions, and case-based deliberations. Topics encompassed epidemiology, clinical manifestations, laboratory diagnostics, genomics, treatment strategies, outbreak investigations, antimicrobial resistance, and One Health perspectives across South and Southeast Asia.

**Results:**

The Congress highlighted expanding geographic recognition of melioidosis across India and neighbouring countries, with reported cases likely representing only a small fraction of the true disease burden. Clinical discussions emphasized marked heterogeneity in presentation and strong associations with diabetes mellitus, environmental exposure, and the monsoon season. Advances in diagnostics included broader implementation of MALDI-TOF mass spectrometry, molecular assays, and rapid diagnostic tests, although significant gaps remain in laboratory capacity, surveillance, and clinical recognition. Genomic investigations demonstrated substantial strain diversity while identifying conserved virulence determinants that may support surveillance and outbreak investigations. Participants also underscored the importance of multidisciplinary collaboration, standardized diagnostic practices, and regional data sharing to improve case detection and patient outcomes.

**Conclusions:**

Melioidosis remains an underrecognized but increasingly important public health challenge in South Asia. Strengthening surveillance systems, expanding diagnostic capacity, improving clinician awareness, fostering regional laboratory networks, and adopting coordinated One Health strategies are essential to reduce morbidity and mortality. The Congress also reinforced the need for sustained advocacy toward recognition of melioidosis as a neglected tropical disease.

## Plain Language summary

Melioidosis is a serious bacterial disease that is common in some tropical regions but remains poorly recognized in many parts of South Asia. Because its symptoms can resemble other illnesses, especially tuberculosis, many patients are diagnosed late or receive the wrong treatment. This can lead to severe illness and death, even though effective antibiotics are available when the disease is identified early.

The 4th South Asian Melioidosis Congress, held in Guwahati, India, in November 2025, brought together experts from several countries to discuss the latest research and practical experience in diagnosing, treating, and preventing melioidosis. The meeting also explored how the disease affects both humans and animals and how environmental factors, such as rainfall and exposure to contaminated soil and water, contribute to infection.

This article summarizes the main messages from the Congress rather than presenting a new research study. It highlights the growing recognition of melioidosis across South Asia, recent improvements in laboratory testing and genomic research, and the continuing challenges faced by healthcare systems, including limited diagnostic facilities, low awareness among clinicians, and weak disease surveillance.

The discussions concluded that many cases of melioidosis are still likely to go undetected. Improving awareness, expanding access to reliable laboratory diagnosis, strengthening surveillance, and encouraging collaboration between clinicians, laboratories, veterinarians, environmental scientists, and public health authorities are essential to reduce illness and deaths. The Congress also called for greater international recognition of melioidosis as a neglected tropical disease to support research, policy development, and investment in better diagnosis and patient care.

## Introduction

Melioidosis, caused by *Burkholderia pseudomallei*, is a severe tropical infection with diverse clinical manifestations and high mortality if untreated. Endemic in Southeast Asia and Northern Australia, recent evidence highlights its growing burden in South Asia, particularly India, Bangladesh and Sri Lanka. ^1, 2^ Despite its significant morbidity and mortality, melioidosis remains under-recognized and under-reported, often misdiagnosed as tuberculosis, pneumonia or other bacterial infections. ^3^ A series of meetings dedicated to raising awareness, fostering collaborations, and advancing research on melioidosis in South Asia (South Asian Melioidosis Congresses - SAMC) was initiated in Manipal, India in November 2015. The fourth and most recent SAMC was held in Guwahati, Assam in November 2025.

## Methods

### Congress design

This evidence synthesis is based on the proceedings of the 4th SAMC, held on 26–27 November 2025 in Guwahati, Assam, India. The Congress brought together more than 400 clinicians, microbiologists, epidemiologists, public health professionals, researchers and policymakers from South Asia and other endemic regions to discuss advances in melioidosis research, diagnosis, clinical management, surveillance and One Health initiatives. The meeting was organized by the South Asian Melioidosis Network in collaboration with regional academic and public health partners. Holding the Congress in Guwahati, Assam—a north-eastern state of India experiencing a rising number of reported cases of melioidosis over the past 2–3 years—was strategically important for enhancing local awareness and capacity. The region has seen increased recognition of melioidosis in recent years due to active surveillance and improved laboratory networks in selected centres, highlighting the need for collaboration to extend across the whole region and eventually country. The two-day event fostered dialogue among participants, facilitating knowledge exchange across clinical, laboratory, environmental and public health domains.

### Data sources

Scientific evidence included in this report was synthesized from all educational and scientific activities conducted during the Congress. Data sources comprised:

• invited lectures;
• keynote and plenary presentations;
• oral scientific presentations;
• poster presentations;
• panel discussions;
• expert consensus sessions; and
• discussions documented in the official Congress abstract book, which can be accessed online ( https://bit.ly/4fjT64o).

### Selection of content

All scientific sessions presented during the Congress were systematically reviewed. Information from multiple presentations addressing similar topics was consolidated, and duplicate or overlapping content was removed to avoid redundancy. The remaining material was examined independently by the authors to identify major scientific themes representing the principal advances and regional priorities discussed during the meeting.

### Data synthesis

The synthesized evidence was organized thematically using a narrative evidence synthesis approach. Scientific content was grouped into the following predefined domains:

• Epidemiology
• Clinical manifestations
• Diagnostics
• Genomics
• Therapeutics and clinical management
• Outbreak investigations and public health
• One Health perspectives
• Future priorities and research directions

This structured thematic synthesis enabled integration of findings across presentation formats and expert discussions, providing a comprehensive and reproducible summary of the current state of melioidosis research, clinical practice and public health priorities in South Asia.

## Results

### Historical and global perspective

The Congress opened with a plenary lecture by Dr David Dance on the historical context of melioidosis in South Asia. ^4^ The first documented South Asian case was reported from Ceylon (Sri Lanka) in 1927, followed by cases in India (1953) and Bangladesh (1964). Nepal initially reported imported cases in 2005, with indigenous cases emerging only in 2019. To date, no indigenous cases have been reported from Pakistan, Bhutan or Afghanistan, underscoring significant gaps in regional surveillance. Early microbiological investigations, notably by Alfred Whitmore and C. S. Krishnaswami, laid the foundation for understanding the epidemiology and pathology of *B. pseudomallei.* In a poignant souvenir write-up, Dr Dance highlighted “The story of Dr C. S. Krishnaswami,” described by his granddaughter as intelligent, diligent and deeply sincere. ^5^ By 1910, Krishnaswami had assumed sub-charge of the Pathological and Post-Mortem Department at Rangoon General Hospital, working closely with Whitmore. His meticulous post-mortem examinations likely provided the first pathological insights into melioidosis, although formal recognition came later. Dr Dance concluded by emphasizing that reported cases represent only the “tip of the iceberg,” advocating for strengthened surveillance and national mandatory notification.

Dr Bart Currie presented global updates, predicting expansion of melioidosis due to climate change, rising sea temperatures, and increased rainfall. He reviewed diagnostic and therapeutic advances while highlighting persistent gaps in diagnostics, infrastructure, therapeutics, funding and global partnerships that demand renewed collective commitment. ^6^

### Regional epidemiology

Dr Chimanjita Phukan presented an overview of melioidosis in India, highlighting emerging outbreaks, diverse clinical manifestations, associated co-morbidities and the importance of a One Health approach. ^7^ She demonstrated year-wise publication trends, state-level case distributions and key risk factors, emphasizing persistent misdiagnosis. Melioidosis is no longer restricted to southern India, with increasing reports from northeastern states, including Tripura, Assam, Manipur and Meghalaya, reflecting broader environmental exposure and improved recognition. ^8, 9^ Dr Saika Farook described enhanced soil culture methods in Bangladesh, linking environmental surveillance with human disease. ^10^ Dr Enoka Corea outlined Sri Lanka’s national microbiology network and surveillance framework, noting a low but rising incidence. ^11^ Dr Trinh Thanh Trung highlighted Vietnam’s integrated clinical–environmental data, including paediatric trends from 2015–2019. ^12^

### Clinical spectrum

Melioidosis is increasingly recognized as a disease of remarkable clinical heterogeneity, in which clinical suspicion remains central to diagnosis, particularly in endemic regions. Across poster and oral presentations, the extensive variability in organ involvement and disease severity reinforced the protean nature of *B. pseudomallei* infection. Pulmonary disease emerged as the most common presentation, ranging from consolidation, infiltrates and pleural effusions to severe pneumonia. However, a broad spectrum of extrapulmonary manifestations was also highlighted, including deep organ abscesses, neuromelioidosis, osteoarticular disease and fulminant septicaemia, underscoring that no single clinical phenotype is pathognomonic for melioidosis. ^13– 15^

Paediatric melioidosis, traditionally under-recognized, received focused attention through cases presented by Dr Rashmi Ranjan Das, involving neonates and infants up to 10 months of age with multisystem disease. These findings reflected increasing detection in Odisha and emphasized the need for heightened clinical vigilance even in very young age groups. ^16^ Numerous clinical vignettes illustrated the invasive and recurrent nature of the disease, including recurrent scalp swellings, infected cysts, and deep organ abscesses—particularly involving the spleen, liver and prostate. ^17^ Paravertebral ^18^ and intracranial abscesses ^18^ were also documented, with some intra-abdominal abscesses occurring in patients with sickle cell disease, highlighting host susceptibility. Post-operative wound infections and pyogenic infections following minor cutaneous trauma further demonstrated the organism’s ability to exploit trivial portals of entry.

Musculoskeletal involvement was frequently reported, including septic arthritis—occasionally polyarticular ^19^—osteomyelitis, intramuscular abscesses and chronic sinus tract formation. ^20^ Severe systemic disease, particularly community-acquired sepsis, remained a major cause of mortality. A consistent seasonal association with monsoon rainfall was documented across Sri Lanka and multiple Indian regions, emphasizing environmental exposure as a key driver of disease incidence. ^21– 26^ Epidemiologically, the first isolation of *B. pseudomallei* from humans in Yadadri Bhuvanagiri district of Telangana represented an important milestone, aligning with broader national trends linking disease occurrence to diabetes mellitus and monsoon exposure. ^27^

Across regions, diabetes mellitus and hypertension emerged as dominant comorbidities, with middle-aged male farmers from rural settings disproportionately affected. ^28^ Recurrent abscesses unresponsive to empirical antibiotics in diabetic patients were emphasized as a key clinical red flag. ^29^ Melioidosis in immunocompromised hosts—including patients with acute myeloid leukemia and those developing secondary hemophagocytic lymphohistiocytosis—often followed an aggressive course with significant diagnostic challenges. ^30^ Prolonged hospitalization was frequently complicated by secondary infections, affecting nearly 40% of patients, with *Candida* spp., *Klebsiella pneumoniae* and *Pseudomonas aeruginosa* predominating. ^31^

Neuromelioidosis remained one of the most severe and diagnostically challenging manifestations, characterized by focal deficits, encephalopathy, cranial nerve palsies and characteristic neuroimaging findings. ^32^ Diagnostic overlap with tuberculosis, leptospirosis, scrub typhus, viral encephalitis and lepra reactions frequently delayed recognition, occasionally exacerbated by corticosteroid-induced immunosuppression. ^33^ Collectively, these presentations reinforced the need for sustained clinical suspicion, timely imaging, early microbiological confirmation and prompt source control to improve outcomes in this often-misdiagnosed disease.

### Diagnostic advances

Recent discussions provided a cohesive overview of both major advances and persistent challenges in the diagnosis and management of melioidosis, emphasizing that technological progress must be matched by clinical awareness and systems-level preparedness. Dr David Dance opened the session with updates on the InBios AMD Plus rapid diagnostic test, including progress toward US FDA approval. He highlighted the limitations of conventional culture-based diagnostics, particularly delayed growth and reduced sensitivity, and underscored the growing promise of lateral-flow immunoassays as tools not only for culture confirmation but also for direct detection of *B. pseudomallei*, potentially enabling earlier diagnosis and treatment initiation.

The clinical and immunological complexity of melioidosis was explored by Dr Madhumita Priyadarshini Das, Dr Susanna Dunachie and Dr Chiranjay Mukhopadhyay, who examined the interplay between host comorbidities, immune responses and survival outcomes. ^34– 36^ Particular emphasis was placed on immune dysregulation in diabetes mellitus—the most significant risk factor for melioidosis—and how altered innate and adaptive immunity contributes to disease severity, persistence and relapse. These insights were linked to ongoing efforts to identify vaccine targets, reinforcing the central role of immunological research in future prevention strategies.

From a laboratory and public health perspective, Dr Narisara Chantratita emphasized the urgent need for integrated laboratory networks to support timely diagnosis, serosurveillance and molecular testing in endemic regions. ^37^ Complementing this, Dr Debkishore Gupta demonstrated the utility of MALDI-TOF mass spectrometry for accurate identification of *B. pseudomallei*, noting that diagnostic reliability depends critically on inclusion of the organism in reference databases. ^38^

Despite these advances, misdiagnosis remains a major obstacle. Tropical isolates are frequently misidentified as *B. cepacia* complex or other species. ^39^ Diagnostic yield can be significantly improved through selective media such as Ashdown agar and CVC-50 broth, particularly for deep-seated and atypical infections including necrotizing fasciitis, diabetic foot infections, psoas abscesses and cervical lymphadenopathy. ^40^ Non-invasive specimens, including urine, were shown to have diagnostic value when incubated for prolonged periods, offering practical advantages in resource-limited settings. ^41, 42^ Automated systems may misidentify *B. pseudomallei* as *Aeromonas* spp., reinforcing the importance of close clinical–laboratory correlation. ^43^

To address these gaps, Dr Ivo Steinmetz presented multiplex serological assays using minimally invasive samples to detect both infection and exposure. ^44^ Immunoproteomic analyses identified multiple candidate antigens, forming the basis for point-of-care platforms supported by smartphone-based imaging and web-enabled interpretation. Advances in serology were further discussed, including the Hcp1-ICT, though variable performance across regions highlighted the need for local validation. ^45^ Molecular diagnostics demonstrated higher detection rates than culture, yet speakers emphasized that improved outcomes rely primarily on early suspicion and timely use of available diagnostics. ^46, 47^

Despite expanding diagnostic options, mortality remains high at 20–30%, particularly among patients receiving corticosteroids, underscoring the imperative for early diagnosis, appropriate antimicrobial therapy and integrated care pathways.

### Therapeutic advances

Clinical management sessions provided a pragmatic overview of melioidosis treatment, reiterating that optimal outcomes depend on timely diagnosis, appropriate antimicrobial selection and strict adherence to prolonged therapy. The standard of care remains a two-phase regimen comprising an intensive phase with ceftazidime or meropenem, followed by eradication therapy with trimethoprim–sulfamethoxazole, with or without doxycycline. ^48– 50^ Deviation from this structured approach was consistently linked to relapse and mortality. Special attention was given to patients with chronic kidney disease, in whom renal impairment complicates dosing and worsens outcomes, necessitating individualized regimens and close monitoring. ^51^ Dr Umang Agrawal emphasized that eradication therapy of at least 12 weeks is essential, as shorter courses are strongly associated with recurrence. ^52^ Early microbiological confirmation, combined meropenem–cotrimoxazole therapy in severe disease and completion of both treatment phases were highlighted as key determinants of survival. ^53^ Encouraging susceptibility data from Central India supported the continued efficacy of first-line agents. ^54^ The importance of timely source control and multidisciplinary supportive care was repeatedly emphasized as central to successful management. ^50^

### Outbreak investigations

Dr R. Prabu presented a detailed account of the 2023 neuromelioidosis outbreak in Tamil Nadu, underscoring the critical role of outbreak investigation and molecular epidemiology in melioidosis control. The cluster of neuromelioidosis cases was traced to contaminated saline used in a dental clinic, and whole-genome sequencing confirmed strain identity across clinical and environmental isolates, conclusively establishing a common source. ^55^ This investigation highlighted how lapses in infection control, even in outpatient settings, can result in severe outcomes when highly pathogenic organisms such as *B. pseudomallei* are involved.

The MISSION Melioidosis initiative, presented by Dr Harpreet Kaur, represents a coordinated national effort to address gaps in recognition, diagnosis and reporting in India. Implemented across 13 sites, the program emphasizes enhanced surveillance, laboratory capacity strengthening and structured training, leading to improved case detection, standardized diagnostics and strengthened regional response networks. ^56^ Complementary environmental surveys from southern Thailand revealed *B. pseudomallei* in 25–26% of soil and water samples, predominantly in non-agricultural areas, and identified novel variants, including *B. thailandensis* ST2063, highlighting unexpected genetic diversity. ^57^

From a broader One Health perspective, Dr Karine Laroucau discussed the related disease glanders in equids, emphasizing its zoonotic relevance and diagnostic overlap with melioidosis due to serological cross-reactivity. ^58^ Collectively, these sessions reinforced that effective melioidosis control requires integration of outbreak investigation, molecular tools and One Health strategies across human, animal and environmental interfaces to prevent transmission and mitigate future outbreaks.

### Genomic epidemiology

*B. pseudomallei* possesses a large bipartite genome (~7.2 Mb) with marked functional compartmentalisation. Chromosome 1 encodes core housekeeping and essential metabolic functions, while chromosome 2 is enriched for accessory genes mediating environmental adaptation, virulence and antimicrobial neutralisation. The species exhibits an open pangenome, comprising a conserved core of ~4,500 genes and a variable accessory component that includes genomic islands, mobile elements and virulence-associated factors, underpinning its ecological adaptability and clinical heterogeneity.

Genome-wide association and burden analyses integrated with transcriptomic datasets have increasingly elucidated genetic determinants of disease severity and outcome. Dr Claire Chewapreecha presented landmark studies from Northeast Thailand identifying bacterial and host variants linked to distinct clinical phenotypes, with multi-omic integration providing mechanistic insights and candidate prognostic biomarkers. ^59^ Addressing limited Indian genomic data, Mr Venkat Abhiram Earny analysed ~200 Indian isolates under the MISSION initiative, revealing extensive genetic diversity, region-specific genomic islands and novel sequence types, while core virulence determinants remained conserved. ^60^ These findings inform surveillance, diagnostics and public health strategies.

## Expert panel discussions

### Topic: “Inclusion of melioidosis as a neglected tropical disease”

A dedicated panel strongly advocated for formal recognition of melioidosis as a neglected tropical disease (NTD), highlighting its substantial yet underappreciated global burden of an estimated 165,000 cases and 89,000 deaths annually. ^1^ Persistent underdiagnosis and weak surveillance, particularly in endemic low- and middle-income countries, were identified as major barriers to visibility and control. Environmental drivers such as soil exposure, rainfall and flooding further complicate disease epidemiology amid climate change. Panellists emphasized that NTD recognition could catalyse improved surveillance, research funding, diagnostics, clinical training and policy prioritization. The consensus was that formal NTD status is essential to reducing misdiagnosis, mobilizing sustained resources and addressing the unacceptably high morbidity and mortality associated with melioidosis.

### Topic: “Hunting the entire iceberg”

Dr Bart Currie and colleagues highlighted persistent misdiagnosis and substantial underreporting of melioidosis in India, emphasizing that the true disease burden remains underestimated. They advocated for systematic surveillance spanning both rural and urban settings, cautioning that a narrow focus on traditional rural risk groups may overlook cases in peri-urban and urban populations. Participants stressed the need for strong state-level reporting, active public health engagement and cross-sectoral collaboration involving veterinary, agricultural and environmental sectors, reflecting a One Health approach. Experiences from North-eastern India demonstrated how improved clinician and laboratory awareness rapidly enhanced case detection. Integration of key host risk factors, particularly diabetes mellitus and chronic kidney disease, into surveillance and clinical algorithms was emphasized to improve early diagnosis and prevention.

### Topic: “One Health and regional challenges”

A panel discussion on One Health approaches highlighted the interconnectedness of human, animal and environmental health in Northeast India, framing melioidosis as a disease requiring integrated, cross-sectoral solutions. Environmental exposure, animal reservoirs and human susceptibility were shown to jointly shape transmission dynamics in this region. Key challenges included limited BSL-3 laboratory capacity, delayed clinical suspicion, diagnostic gaps and inadequate integration of melioidosis into routine public health reporting. Experts emphasized the need for coordinated action across human health, veterinary and environmental sectors, alongside strengthened surveillance, improved data sharing and targeted risk communication. Such One Health interventions were identified as essential for early detection, prevention and reduction of melioidosis burden in Northeast India.

## Discussion

Melioidosis has emerged from being considered a geographically restricted disease of Northern Australia and Southeast Asia to one increasingly recognized across South Asia. The scientific evidence presented during the 4th SAMC provides compelling support that the region is entering a new phase in which improved surveillance, enhanced diagnostic capacity, environmental investigations, and greater clinical awareness are revealing a substantial burden that has historically remained hidden. Rather than reflecting a recent emergence of *B. pseudomallei*, the expanding number of reported cases likely represents improved recognition of an infection that has long been underdiagnosed and frequently misclassified as tuberculosis, pyogenic bacterial infections, or culture-negative sepsis. The Congress reinforced the concept that reported cases remain only the “tip of the iceberg,” emphasizing that the true burden across South Asia is almost certainly considerably greater.

Several factors explain why South Asia is becoming an increasingly important hotspot for melioidosis. The region possesses favourable environmental conditions, including tropical and subtropical climates, heavy seasonal rainfall, extensive rice cultivation, and widespread soil and surface-water exposure, all of which facilitate environmental persistence of and exposure to *B. pseudomallei.* Rapid urbanization, changing agricultural practices, flooding, and climate variability may further increase opportunities for human exposure. Simultaneously, South Asia has witnessed an alarming increase in diabetes mellitus, chronic kidney disease, and other chronic illnesses that predispose individuals to severe melioidosis. Throughout the Congress, diabetes consistently emerged as the dominant host risk factor across multiple countries and clinical settings, reaffirming observations from previous epidemiological studies. Increasing recognition of cases from Northeastern India, Bangladesh, Sri Lanka and other previously under-recognized areas suggests that the disease is geographically far more widespread than earlier appreciated and supports predictive modelling studies indicating extensive environmental suitability throughout the region.

The regional epidemiology discussed during the Congress aligns with global observations while also highlighting several distinctive features. Northern Australia continues to represent the benchmark for comprehensive surveillance, standardized diagnostics and well-established treatment protocols, resulting in improved survival despite high disease incidence. In contrast, most countries in South Asia continue to face delayed diagnosis, limited laboratory infrastructure, and inconsistent surveillance systems. Nevertheless, several important similarities are evident. Clinical manifestations remain remarkably heterogeneous, pulmonary disease predominates, diabetes is the principal underlying risk factor, and seasonal increases consistently follow periods of heavy rainfall. The Congress also highlighted the influence of climate change on the global epidemiology of melioidosis, with increasing rainfall, extreme weather events, and changing environmental conditions expected to expand the geographic distribution of *B. pseudomallei.* These observations position South Asia as a critical region for future surveillance and public health preparedness.

An important advance over previous South Asian Melioidosis Congresses is the evolution from describing isolated case reports toward presenting coordinated regional research programmes. Earlier meetings primarily focused on increasing disease awareness and documenting emerging cases. In contrast, the fourth Congress showcased mature research encompassing multicentre surveillance, environmental microbiology, outbreak investigations, comparative genomics, molecular epidemiology, immunology, diagnostic innovation and One Health approaches. National initiatives such as MISSION Melioidosis demonstrate a transition from passive recognition to active surveillance and capacity building. Likewise, the increasing application of whole-genome sequencing, MALDI-TOF mass spectrometry, molecular diagnostics, multiplex serological assays, and environmental surveillance illustrates substantial technological progress that has occurred within the region. The Congress therefore reflected not only an expansion in scientific output but also increasing regional collaboration and harmonization of research priorities.

Despite these advances, significant knowledge gaps remain. Reliable estimates of disease incidence continue to be unavailable for many endemic regions because surveillance remains fragmented and melioidosis is not routinely incorporated into national reporting systems. Environmental distribution of *B. pseudomallei* remains incompletely characterized across large parts of South Asia, limiting accurate risk mapping and prediction of transmission hotspots. Standardization and regional validation of newer diagnostic tests—including rapid antigen detection assays, molecular diagnostics, and serological platforms—are still required before widespread implementation. Similarly, relatively limited genomic information is available from South Asian isolates compared with Southeast Asia and Australia, restricting understanding of bacterial evolution, transmission dynamics, and regional diversity. Further investigation into host immune responses, vaccine development, antimicrobial resistance mechanisms, long-term clinical outcomes, and relapse remains necessary to improve patient management and prevention strategies.

The clinical implications of the Congress are substantial. The diversity of clinical presentations presented throughout the meeting reinforces that melioidosis should no longer be considered a rare or geographically restricted disease. Clinicians practising in tropical regions should maintain a high index of suspicion in patients presenting with severe community-acquired sepsis, recurrent abscesses, pneumonia, osteoarticular infections, neurological disease, or unexplained febrile illness, particularly among individuals with diabetes, chronic kidney disease and those on high or prolonged corticosteroid therapy. An important clinical message emerging from the congress was the need to consider melioidosis in patients with suspected pulmonary tuberculosis who are acid-fast bacilli (AFB) smear-negative and *Mycobacterium tuberculosis* culture-negative, particularly when radiological findings mimic tuberculosis, constitutional symptoms persist despite empirical anti-tubercular therapy, or patients have risk factors such as diabetes. Early microbiological confirmation remains essential because delayed diagnosis is strongly associated with increased mortality. The discussions also emphasized that advances in diagnostic technology alone cannot improve patient outcomes unless accompanied by increased clinical awareness, optimized laboratory workflows, timely communication between clinicians and microbiologists, and adherence to evidence-based antimicrobial therapy including prolonged eradication treatment.

From a public health perspective, the Congress highlighted the importance of strengthening laboratory capacity and surveillance networks throughout South Asia. Improved access to selective culture media, MALDI-TOF databases containing *B. pseudomallei*, molecular diagnostics, and regional reference laboratories would substantially enhance case detection. Integration of environmental surveillance with human and veterinary health systems would facilitate earlier recognition of emerging hotspots and outbreaks. The successful investigation of the Tamil Nadu neuromelioidosis outbreak demonstrated the value of combining epidemiological investigation with whole-genome sequencing to identify environmental sources and strengthen infection prevention measures. Such experiences illustrate the practical benefits of coordinated multidisciplinary surveillance systems.

One of the most significant policy discussions during the Congress concerned formal recognition of melioidosis as a neglected tropical disease (NTD) by the World Health Organization. Despite causing an estimated disease burden comparable to many established NTDs, melioidosis remains excluded from the WHO NTD portfolio. Participants consistently argued that official NTD designation would increase global awareness, facilitate research investment, strengthen surveillance systems, improve diagnostic access, and promote international collaboration. Recognition would also support integration of melioidosis into national infectious disease programmes, encourage development of standardized reporting mechanisms, and accelerate funding for diagnostics, therapeutics, and vaccine research. The Congress therefore concluded that WHO recognition should be viewed not simply as a symbolic designation but as an important catalyst for strengthening global and regional responses to this frequently neglected disease.

The meeting also strongly reinforced the relevance of One Health principles. Evidence from environmental investigations, veterinary studies, outbreak analyses, and genomic surveillance demonstrated that understanding melioidosis requires integration across human, animal and environmental health sectors. Environmental reservoirs remain the primary source of infection, while climatic and ecological changes increasingly influence transmission dynamics. Coordinated surveillance across agriculture, veterinary medicine, environmental microbiology and clinical medicine will therefore be essential to reduce disease burden and improve preparedness for future outbreaks.

Future research should prioritize large-scale multicountry epidemiological studies to define the true burden of disease across South Asia. Standardized environmental surveillance programmes should be expanded to improve understanding of ecological risk factors and geographical distribution. Regional genomic surveillance networks are needed to characterize strain diversity, transmission pathways, and emergence of novel lineages. Validation of rapid diagnostics and point-of-care tests across endemic settings should continue, accompanied by implementation studies assessing their impact on patient outcomes. Additional priorities include vaccine development, immunological studies identifying correlates of protection, antimicrobial stewardship, optimization of treatment duration, long-term outcome studies, and evaluation of integrated One Health surveillance strategies. Collectively, these priorities provide a roadmap for translating the scientific advances presented during the Congress into measurable improvements in patient care and disease control across South Asia.

A major strength of this report is that it represents the first integrated regional synthesis of scientific evidence presented at the 4 ^th^ SAMC, bringing together contemporary advances from clinicians, microbiologists, epidemiologists, environmental scientists and public health experts across South Asia and internationally. The multinational participation of globally recognized experts provided broad regional representation and facilitated comparison of experiences across diverse endemic settings. Furthermore, the multidisciplinary scope encompassing epidemiology, clinical medicine, diagnostics, genomics, outbreak investigations, therapeutics, and One Health perspectives offers comprehensive coverage of current scientific developments and emerging priorities in melioidosis.

Several limitations should also be acknowledged. This synthesis is based on scientific presentations delivered during the Congress rather than exclusively on peer-reviewed published literature, and some findings represent preliminary data that may subsequently evolve following full publication. The report is intended as a structured narrative evidence synthesis rather than a systematic review and therefore did not follow formal systematic review methodology. Finally, quantitative meta-analysis was neither feasible nor appropriate because of the heterogeneity of study designs, presentation formats, and outcome measures represented across the Congress.

This balanced interpretation provides a comprehensive overview of current regional knowledge while identifying important priorities for future research, surveillance, and policy development.

## Conclusions and implications

The 4th SAMC emphasized the expanding recognition of melioidosis in South Asia, especially in North-eastern India, where surveillance has revealed increasing case numbers. Key conclusions and recommendations included:

1. the need to enhance surveillance to gain a greater understanding of the true disease burden, ideally by making melioidosis a mandatorily notifiable disease in countries in South Asia,
2. the potential benefits of formal NTD recognition by WHO to catalyse investment and research,
3. strengthening laboratory and diagnostic capacity, including rapid tests and MALDI-TOF,
4. enhanced clinical awareness of diverse manifestations and risk factors, including diabetes and CKD,
5. integration of One Health approaches to link environmental, veterinary and human surveillance, and
6. national initiatives, such as MISSION Melioidosis, providing a framework for surveillance, training and outbreak response.

The Congress reinforced the urgency of improving diagnostic capacity, clinical recognition and public health strategies to reduce melioidosis morbidity and mortality in South Asia. Regional collaboration, supported by global expertise, is essential to uncover the “iceberg” of melioidosis cases and implement effective interventions.

## Ethics approval

Ethical approval was not required because this article synthesizes scientific presentations, conference abstracts, expert discussions, and publicly available literature. The study did not involve the collection or analysis of identifiable patient-level data or any new human participant data.

## Consent from patient

Patient consent was not required.

## Consent for naming individuals

We confirm that all individuals named in the manuscript have reviewed the relevant content and have provided permission for their names to be included in the manuscript.

## Use of Artificial Intelligence (AI) tools

During the preparation of this manuscript, the authors used ChatGPT (OpenAI, GPT-5.5) to assist with language editing, improvement of grammar and readability, refinement of manuscript text, and preparation of submission-related documents. The AI tool was not used to generate scientific data, perform data analysis, interpret results, formulate scientific conclusions, or make editorial decisions regarding the content of the manuscript. All outputs generated by the AI tool were reviewed, verified, and edited by the authors, who take full responsibility for the accuracy, integrity, and originality of the final manuscript.

## Data Availability

No primary datasets were generated or analysed in this study. No underlying datasets are associated with this article. This manuscript is based on scientific presentations, invited lectures, panel discussions, abstracts presented during the 4 ^th^ South Asian Melioidosis Congress (26–27 November 2025, Guwahati, India), and publicly available literature cited in the References. No extended data are associated with this article.
