# Supplementary material for: Melioidosis in South Asia: Scientific Advances, Regional Challenges and One Health Perspectives—An Integrated Research Synthesis from the 4th South Asian Melioidosis Congress
Source: Wellcome Open Res. 2026 Aug 21;11:535. [Version 1] doi: 10.12688/wellcomeopenres.27378.1 (PMC13673074; doi:10.12688/wellcomeopenres.27378.1)
Supplement: Peer review history

# Reviewer report: Reviewer response for version 1

**DOI:** 10.21956/wellcomeopenres.30133.r171292

**Published:** 2026-10-15

**Authors:** Patpong Rongkard

**Affiliations:** 1 Imperial College London, London, UK

This manuscript provides a comprehensive summary of current knowledge on melioidosis, encompassing epidemiology, clinical diagnosis, and public health, as presented at the 4th South Asian Melioidosis Congress (SAMC) in late 2025. The authors emphasize that melioidosis continues to be overlooked, underdiagnosed, and misdiagnosed throughout South Asia. The methodology is robust, incorporating perspectives from local clinicians, scientists, and communities, as well as expert updates. The narrative follows a logical progression from foundational concepts to contemporary perspectives and future directions. Panel discussions advocate for World Health Organization (WHO) recognition of melioidosis as a neglected tropical disease to enhance surveillance, research funding, and policy development. Additionally, a One Health framework integrating human, animal, and environmental sectors is proposed. Persistent challenges include resource limitations and restricted data sharing. Much of the data originates from observational and preliminary findings that have not undergone peer review. The evidence is specific to South Asia and may not be generalizable globally, although the findings reflect the underrecognized burden in other endemic regions, such as Southeast Asia.

Is the work clearly and accurately presented and does it cite the current literature?

Yes

If applicable, is the statistical analysis and its interpretation appropriate?

Not applicable

Are all the source data underlying the results available to ensure full reproducibility?

No source data required

Is the study design appropriate and is the work technically sound?

Yes

Are the conclusions drawn adequately supported by the results?

Yes

Are sufficient details of methods and analysis provided to allow replication by others?

Yes

Reviewer Expertise:

Infectious diseases, melioidosis, immunology, transcriptomics

I confirm that I have read this submission and believe that I have an appropriate level of expertise to confirm that it is of an acceptable scientific standard.

# Reviewer report: Reviewer response for version 1

**DOI:** 10.21956/wellcomeopenres.30133.r165505

**Published:** 2026-09-05

**Authors:** Sylvia Chieng

**Affiliations:** 1 Universiti Kebangsaan Malaysia, Bangi, Malaysia

This paper consolidates presentations from the 4th South Asian Melioidosis Congress (Guwahati, 2025). The thematic structure, which includes epidemiology, clinical presentation, diagnostics, genomics, therapeutics, outbreaks, and One Health, works well for organizing a broad, multidisciplinary meeting into a coherent narrative. It effectively conveys the scale of under-diagnosis across South Asia and the region's links to diabetes and monsoon-driven exposure, along with progress in diagnostics and genomic surveillance. However, since it relies almost entirely on unreviewed conference abstracts rather than peer-reviewed data, and reflects one meeting's priorities, its claims (case burden, NTD advocacy) should be read as expert consensus rather than independently verified evidence.

Is the work clearly and accurately presented and does it cite the current literature?

Yes

If applicable, is the statistical analysis and its interpretation appropriate?

Not applicable

Are all the source data underlying the results available to ensure full reproducibility?

No source data required

Is the study design appropriate and is the work technically sound?

Yes

Are the conclusions drawn adequately supported by the results?

Yes

Are sufficient details of methods and analysis provided to allow replication by others?

Yes

Reviewer Expertise:

Host-pathogen interaction, bacterial genomics

I confirm that I have read this submission and believe that I have an appropriate level of expertise to confirm that it is of an acceptable scientific standard.
